# Investigating Effects of Interactive Virtual Reality Games and Gender on Immersion, Empathy and Behavior Into Environmental Education

**DOI:** 10.3389/fpsyg.2021.608407

**Published:** 2021-07-22

**Authors:** Tosti Hsu-Cheng Chiang

**Affiliations:** Graduate Institute of Mass Communication, National Taiwan Normal University, Taipei, Taiwan

**Keywords:** immersive virtual reality, game-based learning, gender, empathy, environmental education

## Abstract

Environmental concerns are obstacles that all humans should confront. Accordingly, Taiwan has incorporated environmental education into its curriculum guidelines; however, difficulties have been encountered in its implementation because Taiwanese society clings to the concept of credentialism, students cannot focus in class, and course content is too abstract. Virtual reality (VR) techniques have been incorporated into the field of education for years; because they can increase the interestingness of learning and also concretely present matters through gamification, the present study applied VR techniques to resolve the aforementioned difficulties. VR can build environments and situations that would be impossible to visit in the real world, e.g., travel inside a human body, or the physically impossible—the surface of Mars. The aim was to increase students’ immersion in class would generate empathy toward the natural environment and perform actual behaviors to protect it. In terms of the experimental design, gender and teaching methods were defined as independent variables, and observation and questionnaires were conducted and applied to examine students’ immersion, empathy, and behaviors after learning. Participants in the experimental group watched a 3D video through a semi-immersive VR device, whereas those in the control group watched an ordinary video accompanied by an explanation from the teacher. The numbers of male and female students were equally allocated, and the learning outcomes of the two groups were further examined. A two-way multivariate analysis of variance test was conducted to examine the influences of 2(genders) × 2(teaching methods) on students’ immersion, empathy, and actual behaviors and also mainly to inspect whether the differences between genders and teaching methods resulted in a direct influence or interaction. The experimental results revealed that the effectiveness of the application of VR techniques is affected by gender; female students presented more favorable performance in both empathy and actual behaviors. In brief, VR techniques can generally enhance students’ learning outcomes; however, limitations such as the cost of VR devices and material designs should also be considered.

## Introduction

Taiwan’s Grade 1–9 (K-12) Curriculum Guidelines stated that environmental education is the process of concept perception and value clarification; it aims to equip students with the skills and attitude required for exploring and appreciating the interactive relationship between humans, cultures, animals, and physical environments. Furthermore, environmental education requires the application of environmental quality decisions and individuals’ self-developed code of conduct. However, in Taiwan’s education system where credentialism is the main focus, environmental education is neglected despite it being included in the curriculum guidelines. In addition, didactic teaching is the most applied method in Taiwan’s education system; teachers deliver lectures “on stage” while students learn “off stage.” Such a deep-rooted educational method is more efficient, yet it can easily lower students’ willingness to learn because of disturbances or difficulty concentrating, and they may not be able to memorize the content they were taught over a long period of time. Moreover, the contents of environmental education are abstract to the extent that, using a didactic teaching method, teachers have difficulty explaining how the environment is deteriorating, what negative effects can be expected when it becomes harsher, and how people’s lives may be influenced. Therefore, even when students understand the situation of animal extinction or what instant dangers such environmental changes may cause to people, they do not develop a sense of empathy, let alone perform actual behaviors to protect the environment.

Environmental education has never been just a subject for the continuation of further studies; instead, it is a mission that all people should embrace. Accordingly, the present study aimed to provide teachers with a briefer, more concrete, and impactful approach for environmental education using a virtual reality (VR)-based method and story design. The purpose was to make environmental education—long neglected because of its dull and abstract contents—valued; enable students to become more involved in environmental education; and encourage them to further respect, befriend, and protect the Earth.

VR techniques have grown mature, and an increasing number of manufacturers have begun to participate in their research and development. Such techniques have been applied in many fields, such as games, advertisement, military, and education; furthermore, with the development of VR techniques, companies have begun to employ them in industries. VR can build environments and situations that would be impossible to visit in the real world, e.g., travel inside a human body, or the physically impossible—the surface of Mars, Solar system or Universe by National Aeronautics and Space Administration (NASA). The most common example is Disney amusement parks where customers are able to indulge in games in a virtual time and space. Moreover, VR elements have been incorporated into home gaming machines, such as the PlayStation 4, allowing game characters to appear in front of players and encouraging players to immerse themselves in the games. In terms of clinical experiments, doctors have also adopted VR techniques to simulate on-site operations for medical students. This informs students of the difficulties they may encounter during surgery and further reduces surgical risks.

The fast development of VR in education not only increases the playfulness of learning but greatly decreases the cost of enhancing students’ willingness to learn. Additionally, events can be presented in a concrete manner with an appropriate level of gamification, which achieves breakthroughs in conventional didactic teaching and improves students’ learning effectiveness. Hence, the present study employed VR techniques in environmental education, expecting to resolve the problems of conventional didactic teaching (e.g., having low immersion and being dull and overly abstract) and further enhance students’ immersion in class, equip them with empathy toward the natural environment, and encourage them to perform actual behaviors to protect the environment.

The present study designed the following storyline set in Taiwan using VR techniques: “People, believing they can conquer nature and do whatever they desire, developed the Earth without limitations, pumped dirty water into the ocean, and air pollution instead emitted pollution into the air. Polar bears cannot find enough food and attack their own species; polar bear cubs have even become the food of adult polar bears. Dolphins are killed for food, and their smiles at the moment they are captured have become the most ironic joke.

Under the continuous deterioration of the Earth’s environment, icebergs are melting faster and sea levels are rising. Imaging one night when everyone is asleep, the devastated Earth fights back ruthlessly. The oceans takes away their homes and families and washes away the modern civilization they are proud of; Taiwan is instantly flooded and everything is submerged under the ocean—only the top floors of Taipei 101 and the rooftop of the Grand Hotel remain. If we are still capable of changing such circumstances, are you willing to help save Mother Earth, the cradle of all life?”

The gender is often discussed in environmental issues, especially the part of empathy (Arnocky and Stroink, 2010). Females usually have more empathy than males in caring about animals, perhaps because of their natural psychological characteristics. Females have behaviors to protect animals, especially when they encounter disasters. Thus, gender will be added to the experiment as independent variable.

The present study designed a VR game and incorporated the aforementioned educationally meaningful story plot. Students of different genders were informed of the importance of conservation through experiencing the distress and dilemma of polar bears strive when they must find another ice berg to survive. High-quality VR devices were employed to present more authentic game images with a higher frame rate, providing students with a realistic experience and strengthening their senses of immersion during the game. In addition to the students experiencing interactivity, challenge, and entertainment during the game, the present study anticipated that they would develop an in-depth cognition and reflection on the story plot, exhibit improved empathy, and transform the educational content into actual behaviors. Based on the experiment, this study further inspected the feasibility and limitations concerning the association of VR techniques and environmental education. The research questions were as follows:

1. Does students’ immersion increase after taking the VR-based environmental course?
2. Does students’ empathy increase after taking the VR-based environmental course?
3. Do students’ behaviors change after taking the VR-based environmental course?
4. Are students’ learning outcomes of different genders significantly different after they take the VR-based environmental course?

## Literature Review

### K-12 Education: Environmental Education

Environmental education has always been an essential part of people’s daily lives, and Taiwan’s compulsory education has also included such a concept in its curriculum. Environmental concerns and environmental education have gained importance since the United Nations Conference on the Human and Environment in 1972; this was later followed by the proposal of “our common future” by the World Commission on Environment and Development. In 1992, the Earth Summit proposed Agenda 21, which emphasized that environmental education is not only a part of national education but also a part of civic basic knowledge and a joint responsibility.

Recently, the idea of an environmental paradigm defining the interaction between humans and nature has gradually expanded from ecological environment conservation to the adjustment of human society and political systems. People have changed their attitude toward technology and economic development from absolute trust to conditional acceptance, and, in terms of time and space, they have extended environmental protection to concern for the living environment of the next generation and further pursued sustainable development. Their values of nature have transformed from human-centered, self-benefiting thoughts to the appreciation of nature and acceptance of the value of all beings.

Taiwan’s K-12 Curriculum Guidelines stated that environmental education is a process of concept perception and value clarification and aims to equip students with the skills and attitude they require when exploring and appreciating the interactive relationship between humans, cultures, animals, and physical environments; furthermore, it requires the application of environmental quality decision-making and i code of conduct on self-positioning. The purpose of environmental education is to equip students with the following abilities: Environmental awareness and sensitivity, environmental conceptual knowledge, environmental values and attitudes, and environmental action skills and experiences. The implementation principles of environmental education consist of integrity, lifelong education, interdisciplinary integration, active participation in problem solving, a balance between world and local views, sustainable development, and international cooperation.

### Immersive Virtual Reality

The first VR prototype was the Sensorama developed by photographer Morton Heilig in 1956. It was a 3D interactive terminal equipped with somatosensory devices. Aside from a 3D display, it had a stereo speaker, odor generator, and vibration seat. Users could experience six intriguing clips on the device, similar to today’s IMAX 4D theaters. However, Sensorama was not prevalent back then, and the concept of VR did not begin to spread until the creation of Oculus Rift devices.

VR techniques are able to simulate the real-world environment, form a 3D virtual world, and integrate or improve current techniques concerning sound, images, illustrations, and text to provide users with a substitute for multiple senses and an immersive experience.

VR, a type of integrative technique, is a high-level, human–machine interface that offers a perceptive experience that is intimate for humans. From a technical perspective, a VR experience should include three basic features, namely immersion, interaction, and imagination, or 3I. VR devices can further be categorized into three types in terms of their level of immersion: Fully immersive, non-immersive, and semi-immersive systems (Alqahtani et al., 2017).

Users are required to wear data gloves and a helmet while using a fully immersive VR device, and their vision is altered by tracking the movement of their heads. In this type of VR system, the user is surrounded by sounds and images, cutting off their perception of the outside world and enabling them to experience full-body immersion. Such techniques require complete devices and are often pricey, such as car simulators. A non-immersive VR system is usually called a desktop VR system, which does not require any device inputs and displays VR images on the screen. It provides lower senses of presence and interaction yet higher picture quality and comfort, and the cost is low; hence, such devices are often used in education and computer games. A semi-immersive system is also called a hybrid system; in such a system, open desktop VR and data gloves are combined along with the use of a simple display system with a highly immersive physical model. The images are a combination of VR and the real world, and participants can interact using data gloves or helmets and control the system through a computer mouse and keyboard. The present study applied a semi-immersive VR system to its experimental group; participants wore headsets and glasses to watch a video displayed on the screen and interact with a computer using a mouse and keyboard. Participants in the control group watched an ordinary video. Subsequently, the differences in the groups’ immersion, empathy, and actual behaviors after they watched a video on a semi-immersive VR device or an ordinary video were examined.

### VR in Educational Applications

VR has engendered a great reform in education. It can provide an immersive environment that enables students to feel as though they are personally at the scene as well as teachers to be more intuitive in their teaching (Klippel et al., 2019). When describing a historical event, teachers can apply VR devices to provide students with a further understanding of the event. Another example is in an astronomy class, where teachers could present each planet using a virtual environment to ensure students better understand the abstract nature of astronomical space (Johnson-Glenberg, 2018). Furthermore, in a chemistry class, teachers could build a virtual laboratory to introduce chemical reactions generated by each chemical substance along with the experimental equipment and procedures, which is more space-efficient, prevents danger caused by students’ improper operation, and motivates them to learn by making the learning process fun and safe (Edwards et al., 2019).

Furthermore, because of the advancement of smartphones and other devices, students of the younger generation are accustomed to the stimulation of sound and light effects, and thus current teaching methods have become dull to them. Therefore, they may not be able to pay attention in class and present low learning efficiency when teaching materials are monotonous. However, the application of VR techniques in education cannot only improve students’ attention but also their learning outcomes, and thus, they provide teachers with multiple teaching methods (Hamilton et al., 2021). In brief, the benefits of VR techniques for educational purposes are as follows:

#### Increasing Students’ Concentration Span

Average individuals can memorize 20 and 30% of the contents they hear and read, respectively, but up to 90% of the contents through actual operation or imitation. The application of VR techniques establishes a virtual environment that enables students to conduct operations and imitations on their own, effectively enhancing students’ concentration.

#### Overcoming Space Restrictions and Making Actual Operations Easier

In the process of learning, many actual operations are omitted because of space restrictions or funding shortages. For instance, junior high school chemistry teachers must often sacrifice students’ rights to conduct experiments because of the lack of classrooms or materials. Such courses then merely become a theoretical discussion, resulting in a serious discrepancy between theory and students’ perception. If students can enter the virtual environment using VR techniques, they can more easily realize the importance of ecological balance and acquire a more complete understanding of the actual situation.

#### Transforming Teaching From Unilateral to Bilateral Teaching

Through conventional didactic teaching, teachers unilaterally deliver lessons while students passively receive knowledge. However, the application of VR techniques provides students with immersive contents, which encourage them to become involved in learning. “Knowledge” and “actual operation” are associated and mutually interactive, complementing the limitations of didactic teaching and improving teachers’ teaching effectiveness.

### Game-Based Learning

Game-based learning (GBL) is a type of learning approach that combines educational contents with games for users to learn and aims to trigger learners’ motivation and intention in constant learning for enhanced learning effectiveness. Computers and mobiles devices are adopted as equipment when such approach is applied; therefore, most studies have referred to it as digital GBL. Bersin (2017) proposed four new types of digital learning that concern enterprise learning, among which GBL exhibited a sustainable growth of 32%. The main reason for its gained importance and rapid development is that games are applied to stimulate learners’ involvement and motivation, which resolves the disadvantages of conventional teaching and other digital learning methods that cannot attract learners to participate. James Paul Gee elaborated the benefits of GBL in his publication titled *What Video Games Have to Teach Us About Learning*. First, games are designed with an instant feedback function; learners are rewarded for every little effort and gradually given questions. Learners can further grow in confidence by solving the questions repeatedly and developing enthusiasm toward such knowledge. Furthermore, many games are infused with competitive and cooperative elements to strengthen learners’ social skills, which can serve as their essential assets in the future.

Recently, more researchers have discovered the effects of games on their teaching effectiveness, including them facilitating learning motivation, establishing social skills, and adjusting attitudes. Preparation for future learning has therefore become a common content design concept in GBL; using this concept, learners are encouraged to use their acquired knowledge to proactively acquire new knowledge when confronted with unfamiliar problems. Accordingly, the present study asserted that learners can achieve high immersion and generate empathy and practical social behaviors after receiving environmental education through VR GBL.

### Situated Learning

Situated learning contributes to bringing about the relationship between classroom situations and real-life situations outside the classroom. To be specific, it cannot be isolated from its environmental context. Furthermore, knowledge is a type of tool, the value of which cannot be distinctly highlighted if it is not properly used (Brown et al., 1989). When learners are disconnected from reality, their “knowledge” and “action” are separated, and what they acquire is merely “inert knowledge” (Whitehead, 1929), which is difficult to apply in reality. Furthermore, abstract knowledge is difficult to transfer into similar scenarios (Anderson et al., 1996); such knowledge cannot last and may result in cognitive loads in learners’ perception (Anderson et al., 1996).

Accordingly, situated learning highly accentuates the authenticity of learning activities, yet this does not imply that the learning effectiveness of outdoor teaching is superior to that of conventional classroom lectures (Moore and Lackney, 1994). Specifically, learning activities should correspond to actual situations, and such activities possess a certain authenticity when they are meaningful and purposeful when applied to actual situations. Hence, Collins and Frederiksen (1989) compiled relevant literature and defined strategies for situated learning, namely authenticity, intersectionality, connectivity, reflectivity, circularity, and the application of multimedia. These strategies emphasize guiding learners in how they can apply their knowledge or skills to their lives or other scenarios, learn by analogy, and examine the effectiveness through reflection to continually improve. Moreover, selecting and employing appropriate media can enhance learning effectiveness at suitable times.

Situated learning includes numerous practices, including apprenticeship, collaboration, reflection, coaching, multiple practice, the articulation of learning skills, realistic representations, and the application of technologies (McLellan, 1996). Teachers can collocate the different practices and establish a situated learning field that better suits the learners. The present study adopted the said six strategies of situated learning as design concepts and incorporated actual scenes and introspective learning methods to develop a game environment that conform with learners’ preceding life experiences and provide a space that enables instant reflection to make learners fully immersed in the purpose of the present study.

### Flow Theory

Flow theory was proposed by Csikzentmihalyi, a renowned psychologist. He claimed that when individuals indulge in a certain event or activity (e.g., the Internet, sports, and games), they tend to be particularly focused, fully integrate into the activity, and clear all irrelevant thoughts, which is called the “flow state.” When individuals enter such a state, they share a similar experience mode, under which they filter all irrelevant feelings and thoughts, lose their self-consciousness, generate reactions toward only a specific target or concrete responses, and develop a sense of control over the environment. Other researchers explained the flow state as a situation where “individuals are concentrated on an activity and enjoying the fun during the process.” Because of the pleasure derived from immersive experiences, individuals tend to participate in the activity continuously and repeatedly (Csikzentmihalyi, 1990).

Webster et al. (1993) extended Csikzentmihalyi’s (1990) concept and adopted flow theory to examine the interaction between humans and computer information systems. Webster asserted that during human–computer interactions, participants subjectively perceive pleasure and a sense of participation. Events with greater enjoyment generate more positive emotions, and such emotions attract participants to engage in further explorations. Therefore, Webster suggested that a human–computer experience is a type of flow experience that is playful and exploratory. Furthermore, Webster et al. (1993) claimed that a flow experience can be categorized into four concepts: Control, attention focus, curiosity, and intrinsic interest.

Control means that participants can adjust their own operant behaviors using responses generated through interactions with computer information. Attention focus describes how participants filter irrelevant perceptions when involved in a narrow range of activity. Curiosity is derived from all types of novel and tempting stimuli, which trigger participants to feel curiosity. Intrinsic interest is the reason why participants continually indulge in games, and such behaviors are not sustained by their jobs or other intentions but a stronger sense of intrinsic interest, such as the enjoyment of the process of VR interaction and the pleasure produced by the responses. Thus, the flow experience presented by Webster et al. (1993) is inseparable from interactivity and pleasure, which attract individuals to indulge their senses in a certain activity.

In flow theory, challenges and skills are the most essential factors, and participants can only reach their optimal flow experience when these two factors are balanced. Csikszentmihaly indicated that if the challenges exceed participants’ skill levels, participants will become anxious, whereas if challenges are too easy relative to their skill levels, participants may feel bored. These two situations force participants to leave a flow state; therefore, game designers should strike a balance between challenges and skills to ensure participants enter a flow state during their participation.

Csikzentmihalyi and Rathunde (1993) stated nine factors that lead to the development of a flow state: Clear goals, immediate feedback, matched challenge and skills, the merging of actions and awareness, concentration on the task, a sense of potential control, the loss of self-consciousness, an altered sense of potential control, and the autotelic experience. To make players indulge in a game, challenges and rewards are required when the game starts, and the challenges should be difficult enough to trigger players’ interest. Enjoying the game process then becomes the purpose. Players can indulge in the game and play it repeatedly and will not even feel time passing—which is the power of immersion.

## Materials and Methods

### Research Framework

**FIGURE 1:**
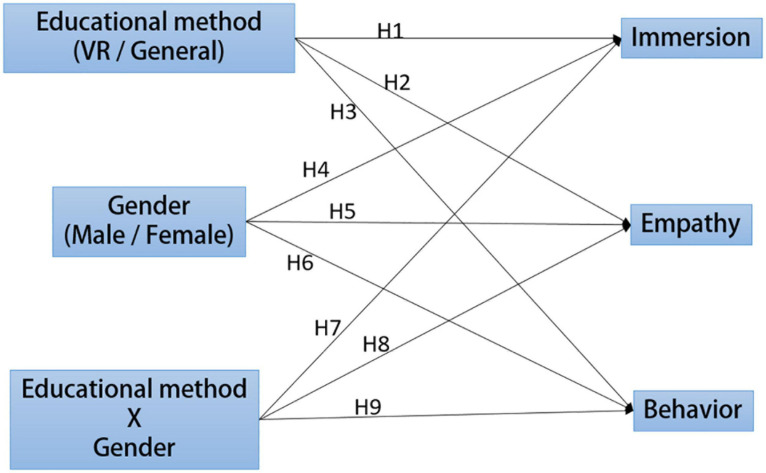
Research framework and assumptions.

The design concept of the developed learning material was based on situated learning theory. The features of VR techniques were incorporated, and the content was designed in accordance with the Grade 1–9 Curriculum Guidelines with the theme of doomsday scenes in Taiwan. Situated learning emphasizes that learners should interact in real situations in society, acquire knowledge in simulated scenarios through activities, and develop a reasonable and meaningful interpretation of the acquired knowledge (Brown et al., 1989). Collins (1994) claimed that situated learning is authentic, connective, intersective, and reflective, and also emphasized that learners can engage in proactive learning, problem identification, and problem solving, eventually applying the knowledge acquired through GBL to their lives. The present study evaluated junior and senior high school students’ level of acceptance toward the implementation of environmental education through situated VR GBL and established a research framework on the basis of situated learning theory, VR techniques, and the technology acceptance model, as displayed in Figure 1. Students’ gender and teaching methods were selected as independent variables, whereas level of immersion, empathy, and actual behaviors were selected as dependent variables.

Based on the research purpose, the present study established the following assumptions:

1. This study discussed whether different teaching methods, compared with conventional ones associating didactic teaching with videos, significantly influence students. It assumed that VR contributes to favorable learning outcomes, and proposed the following hypotheses accordingly:

H1: Different teaching methods significantly influence students’ immersion in environmental education.
H2: Different teaching methods significantly influence the empathy generated in students by environmental education.
H3: Different teaching methods significantly influence the actual behaviors of students generated by environmental education.

2. The learning outcomes of students of different genders can be distinctive. Clarke et al. (2016) indicated that women presented a significantly higher level of empathy and learning motivation than did men; hence, the present study examined whether students’ gender resulted in different learning outcomes through implementing environmental education, and proposed the following hypotheses:

H4: Gender significantly influences students’ immersion in environmental education.
H5: Gender significantly influences students’ empathy generated by environmental education.
H6: Gender significantly influences students’ actual behaviors generated by environmental education.

3. The present study further discussed the interaction between gender and teaching methods, namely whether students of different genders produce distinctive learning outcomes when different learning methods were applied. Accordingly, the following hypotheses were proposed:

H7: The interaction between gender and teaching methods significantly influences students’ immersion in environmental education.
H8: The interaction between gender and teaching methods significantly influences students’ empathy generated by environmental education.
H9: The interaction between gender and teaching methods significantly influences students’ actual behaviors generated by environmental education.

### Experimental Design

#### Story Design

This interactive learning VR game is divided into five scenarios, named saving the ocean, protecting dolphins, protecting polar bears, surviving in the sea, and protecting the Earth.

1. Scene 1:

Humans, a late-coming species, have wantonly destroyed the Earth. They have pumped dirty water into the ocean and wiped out half of marine life. Contaminated gas is directly emitted into the air, and trees that protect the Earth are chopped down to provide merchants with extra space and profits. The user must fight against the contaminated sources to protect the Earth.

**FIGURE 2:**
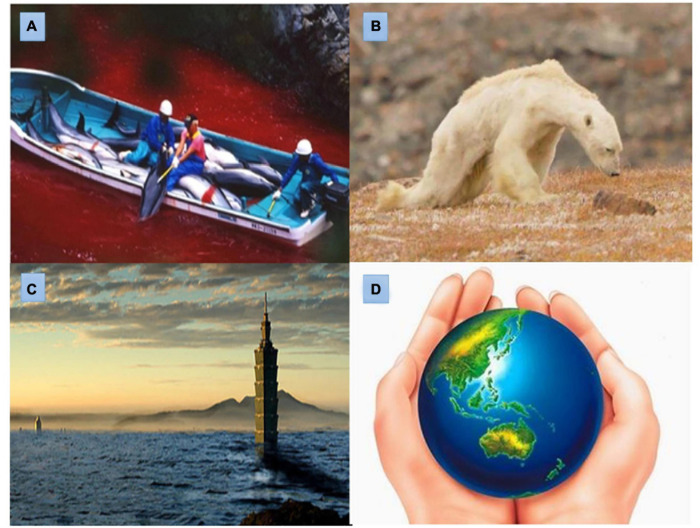
Game scenes. **(A)** The game of guarding Dolphins. **(B)** The game of saving the polar bear. **(C)** The game of the waterworld. **(D)** An environmental game that protects the earth.

2. Scene 2:

Dolphins once regarded humans as friends and welcomed them with smiles, yet they ruthlessly took knives to them, killing them one after another, making their smiles the most ironic joke ever. They care only about their food and the money that fills their pockets, letting rivers be dyed red with dolphin blood (as depicted in Figure 2A). Users as dolphins must escape human killing to save their lives.

3. Scene 3:

Have you ever thought about how the Earth has deteriorated under human destruction? Have you ever imagined that melting icebergs in polar regions would cause polar bears to become skinny (as depicted in Figure 2B) and be forced to eat bear cubs because they cannot find enough food? Can you humans ever picture a penguin standing helplessly on a floating iceberg, gazing toward ice in the distance, and hesitating over whether to die in the freezing water or starve on land? Users as polar bears must find food in the melting iceberg to prevent starvation.

4. Scene 4:

You are not willing to cherish your only home and have chosen to destroy it for a convenient life. If you are reluctant to protect your home, you should know what disasters may come when the Earth perishes. One night when you are sleeping deeply, the levees break, causing water to flood your city and ruin the civilization you are proud of. Those familiar homes are washed away; only the top floors of Taipei 101 and the rooftop of the Grand Hotel can be seen (Figure 2C). However hard you strive, you can only be washed into the flood and float in the water.

5. Scene 5:

If there was still a tomorrow, if everything could start over, would you be willing to protect the Earth, guard your only home, value environmental protection, and conserve the ecology, starting from you and me (Figure 2D)? Together we should build a home for sustainable development and ensure our descendants are left a more favorable environment before everything is too late. This scene is a knowledge-based interactive game, users can pass through interactive questions and answers.

#### System Design

**FIGURE 3:**
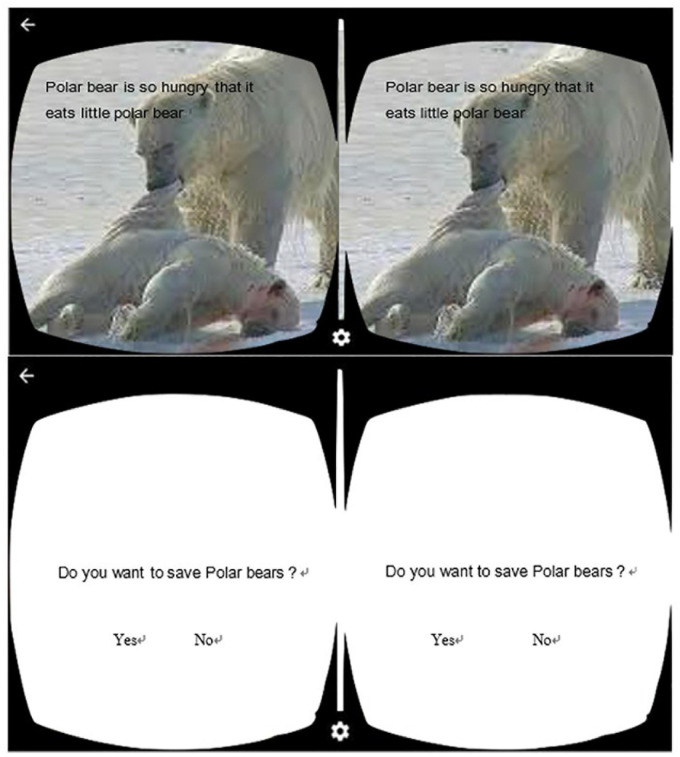
Simulated VR scenes.

The design concept of the game environment was experiential learning, as displayed in Figure 3. Through VR techniques, a sense of presence was created to strengthen concrete experiences, and the game background was designed in accordance with the current environmental situation and the possible disasters to come. A series of devastating images and sad music were applied to immerse students in the virtual environment. Moreover, interactive activities were included in the games; students were asked questions about how they would manage environment- or ecology-related problems, and every choice they made was recorded. Eventually, the present study assigned students to discussions and examined whether they had developed a higher level of empathy toward animals, grown concerned about the environment, and further taken practical actions after taking the VR-based course. The images presented in the VR video consisted of the following four parts:

1. The current situation the Earth is facing: Humans pouring dirty water into the ocean and killing dolphins for food.
2. The ecological problems humans have caused to the Earth: This part enabled students to experience the helplessness of a polar bear standing on a floating iceberg and witnessing polar bear cubs being captured by another polar bear, as displayed in Figure 3.
3. Students’ choices when confronting a series of environmental and ecological problems and the consequences of the different choices they made.
4. Students reflecting on the importance of environmental protection and cherishing the Earth of their own volition.

#### Experimental Procedures

**FIGURE 4:**
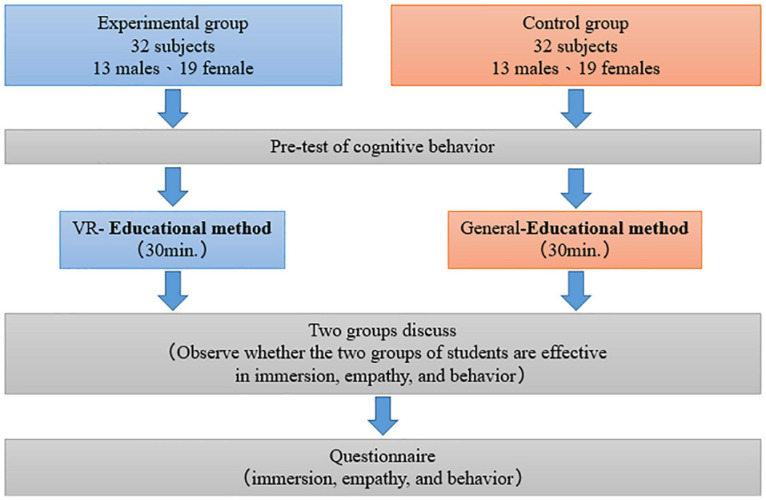
Experimental procedures.

**FIGURE 5:**
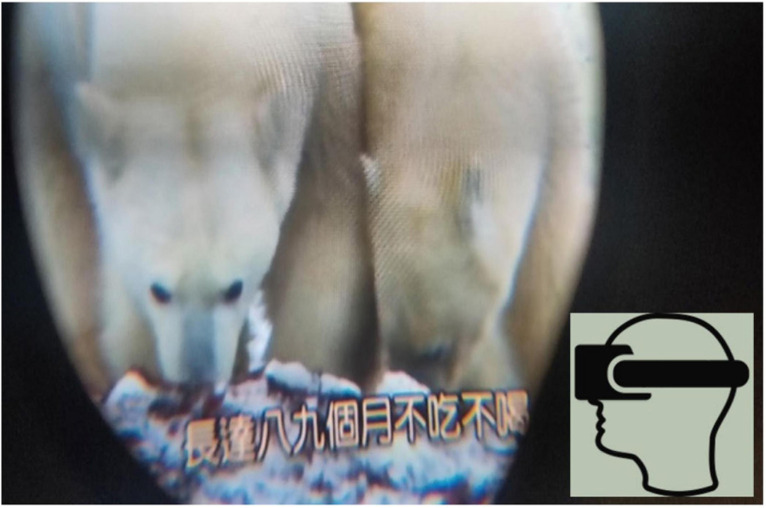
Students in the experimental group watching the 3D virtual reality video.

The 64 participants were evenly divided into experimental and control groups, with 32 participants (13 male and 19 female students) in each group. As displayed in Figure 4, both groups were first given an environmental education agreement questionnaire to evaluate whether different teaching methods are able to influence their empathy and behaviors in terms of the environment. Subsequently, the teaching began separately in both groups for approximately 30 min. The VR-based teaching method was applied to the experimental group (Figure 5), whereas the didactic teaching method used with an ordinary video was employed for the control group. The Extinction of Polar Bears from the Orphans of the Earth series hosted by Pai Hsin-I and produced by Eastern Broadcasting^1^ was adopted as the teaching material of this experiment. Students in the control group watched the ordinary video, whereas those in the experimental group experienced 3D panoramic VR edited by the technical team of a university in Taipei. The effects of the VR images are depicted in Figure 5, and the content was also adapted from the source video to examine whether differences exists in students’ immersion and empathy when different devices were employed. The researchers then asked students in both groups to discuss the course they had just taken and observed the differences in their immersion, empathy, and behaviors toward environmental protection after experiencing the two different teaching methods. Eventually, all students took a questionnaire posttest that evaluated their understanding of the environment and inspected their immersion, empathy, and actual behaviors.

#### Questionnaire Design

The questionnaire was designed using a 5-point Likert scale for measurement, ranging from strongly agree to strongly disagree, to investigate students’ learning effectiveness after using VR devices. Immersion, empathy, and actual behaviors were defined as the three main variables.

The interaction measurement of immersive experiences was adapted from Witmer et al. (2005). Immersion was analyzed through two dimensions, namely the level and tendency of immersion, and the interaction scale comprised 16 questions that examined whether students felt a sense of immersion when taking the VR-based course.

The empathy questionnaire was divided into two scales, individual worries and sense of empathy, which were adapted from relevant questionnaires developed by Mehrabian and Epstein (1972) and Davis (1983). The scales were used to measure students’ empathy and observe whether they became empathetic during the VR-based course and further developed empathy toward the environment and protected species. Eventually, the present study discussed whether empathy was able to stimulate actual behaviors and shifted the course focus to the practice of environmental conservation.

## Results

The present study retrieved 64 questionnaires from 38 and 26 female and male participants, respectively. Reliability and validity tests were first implemented from the aspects of the three variables of immersion, empathy, and actual behaviors based on the data obtained from the questionnaires. Questions concerning the immersion variable were divided into two scales: The level and tendency of immersion. The level of immersion scale examined whether students were isolated from external disturbances when using the VR devices, whereas the tendency of immersion scale concerned students’ emotions and status when playing the game. Questions regarding the empathy variable were also categorized into two scales: Individual worries and sense of empathy; the individual worries scale focused on how the game made users feel, whereas the sense of empathy scale examined whether students could think from the perspective of the Earth after watching the video. Questions related to the actual behavior variable explored whether students were willing to deliver relevant information and protect the Earth after playing the game. The results of the reliability and validity tests in terms of the three variables that the reliability of immersion, empathy, and actual behaviors were 0.84, 0.92, and 0.824, respectively, and the Cronbach’s alpha values of the three variables all exceeded 0.8, which implied that the results of all five scales had high reliability.

Subsequently, the present study implemented a two-way multivariate analysis of variance (MANOVA) test to examine the influences of 2(genders) × 2(teaching methods) on students’ immersion, empathy, and actual behaviors, mainly inspecting whether the differences between genders and teaching methods resulted in different levels of immersion and empathy and different types of behaviors.

The results of Box’s test for equality of covariance matrices (Box M = 19.753, F = 0.992, and *p* = 0.465 > 0.05) are insignificant and does not violate the test. Furthermore, the results of Levene’s test on immersion, empathy, and actual behaviors were F = 1.505, *p* = 0.222 > 0.05; F = 2.246, *p* = 0.092 > 0.05; and F = 1.683, *p* = 0.18 > 0.05, respectively. The *p*-values of all three variables exceeded 0.05, and thus did not violate the homogeneity test. The following analysis is mostly based on the data of Wilks’ lambda.

The results of the MANOVA test on the major influences of the independent variables (i.e., teaching methods and gender) on students’ immersion, empathy, and actual behaviors (Table 1) proved that the dependent variables exhibited significant differences with teaching methods (Wilks’ lambda = 0.217, *F*(3, 58) = 69.911, *p* = 0.000 < 0.0001^∗∗∗^, particle η^2^ = 0.783), gender (Wilks’ lambda = 0.849, *F*(3, 58) = 3.427, *p* = 0.023 < 0.05^∗^, particle η^2^ = 0.151), and also the interaction between teaching methods and gender (Wilks’ lambda = 0.847, *F*(3, 58) = 3.48, *p* = 0.021 < 0.05^∗^, particle η^2^ = 0.236). This suggested that different teaching methods and genders led to different learning effectiveness, and further analysis on the significance of the independent variables to each dependent variable was conducted to clarify which learning performance were affected.

**TABLE 1:** Results of the multivariate analysis of variance test.

**IV**	**Wilks’Λ**	**F**	**df**	**Partial η^2^**	**Sig.**
**Main effect**					
Teaching method	0.217	69.911***	3.000	0.783	0.000
Gender	0.849	3.427*	3.000	0.151	0.023
**Interaction effect**					
Teaching method × Gender	0.847	3.480*	3.000	0.153	0.021

*p < 0.05, ***p < 0.001.

Subsequently, the results of the questionnaire were adopted for a univariant analysis, as demonstrated in Table 2. Teaching methods had a significant influence on immersion, empathy, and actual behaviors, and the results were as follows: *F*(1, 60) = 98.821, *p* = 0.000 < 0.0001^∗∗∗^, particle η^2^ = 0.622; *F*(1, 60) = 153.156, *p* = 0.000 < 0.0001^∗∗∗^, particle η^2^ = 0.719; *F*(1, 60) = 45.623, *p* = 0.000 < 0.0001^∗∗∗^, particle η^2^ = 0.432. The present study inferred that the learning outcomes of students who watched the VR video and those who watched the ordinary video along with didactic instructions were significantly different; therefore, H1, H2, and H3 were valid.

**TABLE 2:** Results of the univariate analysis of variance test.

**IV**		**Immersion**	**Empathy**	**Behavior**
	**df**	***F***	***F***	***F***
Teaching method	1	98.821***	153.156***	45.623***
Gender	1	2.423	5.212*	7.44**
Teaching method × Gender	1	3.631	4.146*	6.943*
Error	60			

*p < 0.05, **p < 0.01, ***p < 0.001.

Moreover, gender did not have a significant influence on immersion [*F*(1, 60) = 2.423, *p* = 0.125 > 0.05, particle η^2^ = 0.039]. But gender had a significant influence on empathy and actual behaviors, and the results were as follows: *F*(1, 60) = 5.212, *p* = 0.026 < 0.05^∗^, particle η^2^ = 0.08; *F*(1, 60) = 7.44, *p* = 0.008 > 0.01^∗∗^, particle η^2^ = 0.11. However, this implied that after receiving environmental education, students of different genders presented a similar level of immersion (H4 was invalid), yet their empathy and willingness to perform actual behaviors exhibited significant differences (H5 and H6 were valid).

In addition, the interaction did not have a significant influence on immersion [*F*(1, 60) = 3.631, *p* = 0.062 > 0.05, particle η^2^ = 0.057], hence, H7 was invalid. However, the interaction between teaching methods and gender had a significant influence on empathy and actual behaviors [*F*(1, 60) = 4.146, *p* = 0.046 < 0.05^∗^, particle η^2^ = 0.065; *F*(1, 60) = 6.943, *p* = 0.011 < 0.05^∗^, particle η^2^ = 0.104], H8 and H9 were valid. Thus, given that the interaction between teaching methods gender and poses significant influences, the present study focused on examining their joint influences on students.

As previously stated, when the VR teaching method was applied, both male and female students presented a higher level of immersion without a significant difference as well as developed greater empathy, although the significance was higher for female students than it was for male students. Moreover, female students exhibited a more significant level of empathy than did males only after watching the ordinary video; therefore, when watching the VR video, they were anticipated to outperform males, who did not exhibit increased empathy after watching the ordinary video. In other words, differences in empathy and actual behaviors existed between the genders, yet both male and female students developed an increased level of empathy through watching the 3D VR video despite the differences between them. Female students exhibited a more significant improvement in actual behaviors, which widened the gap between genders; however, male and female students had a similar level of immersion, and therefore no significant difference was discovered between the two genders after the VR teaching method was applied.

According to the experimental results, the effectiveness of the VR teaching method was influenced by gender; nevertheless, VR techniques are assistive in improving partial effects on students’ learning. This explained that the 3D space simulated by VR techniques can authenticate and concretize the sound and images of conventional videos, making users feel immersed through the extension and substitution of multiple senses. Students using the VR devices felt immersed in the virtual environment, generated empathy toward the threats facing the ecosystem, and further increased their willingness to perform actual behaviors to protect the environment.

## Discussion and Conclusion

Because the environment is gradually deteriorating but the situation has not drawn much attention, the present study aimed to provide students with immersive experiences in combining with scenes, stories, and educational meanings through VR interactions and an environmental education video, further influencing their cognition, attitude, and behaviors toward environmental conservation. A questionnaire survey was conducted to examine whether the association of VR techniques and environmental education was effective. The results revealed that compared with students who received conventional didactic teaching and viewed an ordinary video, the students who experienced the 3D VR teaching approach presented a significant difference in terms of learning absorption; furthermore, those who experienced the VR teaching approach performed with significantly higher immersion during the 30-min course. In addition, students produced a different level of empathy toward the environment due to different teaching methods. Those who took a VR-based course exhibited greater empathy toward the survival of protected species, which generated their desire to help the animals, protect global environments, and increase their awareness of the importance of global environmental conservation.

According to Clarke et al. (2016), females present higher levels of empathy and learning motivation compared with males. Considering that gender was an influential factor on the experimental results, the present study defined gender as an independent variable. The results demonstrated that female students were superior to male students in terms of empathy and actual behaviors independent of the control and experimental groups, whereas no significant differences were discovered in their immersion. This study discovered that all participants presented a similar level of immersion because they were given the same equipment, yet female students were more empathetic and proactive in their behaviors because they readily generated emotional fluctuations toward ecological or animal-related environmental concerns. This does not contravene the fact that the 3D VR teaching approach is more effective; however, to equip male students with excellent empathy and proactive behaviors, the differences between male and female students should be overcome when environmental education courses are designed.

In accordance with the aforementioned experimental results, the teaching method involving a 3D VR video indeed resulted in superior learning effectiveness compared with the conventional method involving an ordinary video; the results of the present study indicate a novel teaching method for environmental education. The reason behind the success can be attributed to the features of VR. The VR devices adopted by the present study were a helmet, headset, and mouse in combination with a desktop and immersive VR systems, which is categorized as a semi-immersive VR system. Such devices can block exterior disturbances to make the VR-based learning situation more concrete and authentic, enabling learners to become naturally immersed when watching a video. During the experiment, the learners interacted with animals or made choices from a first-person perspective, and such human–computer interaction adds playfulness to the learning process and also solves the problem of didactic teaching being too abstract and dull. Additionally, learners sensed the difficulties encountered by the animals and natural environments through photorealistic operations; therefore, they could better imagine the real-world situation, reflect on their own behaviors, and relate to other relevant environmental concerns. The aforementioned features of VR correspond to 3I (immersion, interaction, and imagination); accordingly, compared with the 2D bystander perspective produced when watching an ordinary video, VR techniques enable learners to be easily immersed, stimulate their empathy, and encourage them to perform actual behaviors after learning, which is more crucial in terms of environmental education.

In the past, researchers once employed VR techniques in natural science, attempting to break the limitations of time and space, enabling students to roam in human bodies and take a closer look at the abstract concept of cells and organs taught by teachers. Such techniques were also applied in the field of humanities, allowing students to experience the locations where ancient literati visited. However, environmental education, which requires the acquisition of knowledge and the cultivation of empathy and actions, differs from general subjects. Under the accelerated deterioration of the environment, repetitive propaganda in class has become clichéd. Memorizing slogans such as “Turn of the lights when leaving” and “Take public transportation more often” is indeed simple for students, yet making them perform actual behaviors is not easy. The Grade 1–9 Curriculum Guidelines in Taiwan specifically declared the development of “environmental action skills” to be one of the purposes of the environmental education course. With the development of technology, VR techniques have been applied in different industries. Therefore, the present study adopted such techniques for its experiment and proved that the application of VR in environmental education stimulates the advantages and potential of empathy and actual behaviors. If VR becomes prevalent and lower in cost, it will indeed serve as a great help in environmental education. Other subjects that require students to take practical actions, including gender equality and human rights education, can also benefit from using VR technologies. Although students’ learning effectiveness in these subjects cannot be evaluated through examinations, they are universal values and basic competences that all humans should hold and should be valued accordingly.

Aside from the success of applying VR techniques, the design of the content is equally essential. The present study adopted the Extinction of Polar Bears from the Orphans of the Earth series produced by Eastern Broadcasting as the main material in both the experimental and control groups. The content in both groups was identical, and the purpose of the experiment was to investigate the features of VR devices; however, the fact that the application of different devices has significant influences is not to be neglected. The interactive questions in the game and the 3D effects of the images replaced the supplementary information method supported by didactic teaching. Compared with conventional courses, VR devices seem to further substitute a teacher’s position, which raises the concern of whether teachers will be absent from classrooms in the future. From an optimistic perspective, teachers will not be replaced and may instead feel less burdened. They can import the teaching content in VR devices in advance and interact with students through games; consequently, VR teaching materials can be applied repeatedly in different classes, and students’ immersion and concentration can also be enhanced. Nevertheless, teachers should learn to exploit VR techniques to avoid being replaced or surpassed by VR devices.

The present study accentuated the differences between devices; hence, VR and ordinary videos were selected for the experimental and control groups, respectively. Future studies that examine the influences of VR devices on education are recommended to reduce the differences between devices to seek a consistent foothold between the two groups, which will guarantee a further discussion on the influences of factors such as interaction or scenario designs on environmental education. The present study made much effort to balance the gender ratio in the experimental and control groups, yet both groups had six more female students than male students, which revealed a lack of rigor. Furthermore, the actual behaviors generated by empathy could only be examined through observation during the student discussions and questionnaire survey, and such results reflect an instant effect within the short term. If long-term and continuous behaviors are to be analyzed, subsequent observations or another questionnaire survey are required after a period of time. However, the present study could only define the instant learning effect as the main research variable because of the lack of time and the inconvenience of further observation. Follow-up studies are recommended to analyze long-term learning effects to replenish studies in relevant fields.

## Data Availability Statement

The raw data supporting the conclusions of this article will be made available by the author, without undue reservation.

## Author Contributions

The author confirms being the sole contributor of this work and has approved it for publication.

## Conflict of Interest

The author declares that the research was conducted in the absence of any commercial or financial relationships that could be construed as a potential conflict of interest.

## Footnotes

1 https://www.youtube.com/watch?v=pE4-PKfbNo8
